# Clinical Experience of Patients Receiving Doripenem-Containing Regimens for the Treatment of Healthcare-Associated Infections

**DOI:** 10.1371/journal.pone.0167522

**Published:** 2016-12-01

**Authors:** Chien-Ming Chao, Chi-Chung Chen, Hui-Ling Huang, Yin-Ching Chuang, Chih-Cheng Lai, Hung-Jen Tang

**Affiliations:** 1 Department of Intensive Care Medicine, Chi Mei Medical Center, Liouying, Tainan, Taiwan; 2 Department of Medical Research, Chi Mei Medical Center, Tainan, Taiwan; 3 Department of Health and Nutrition, Chia-Nan University of Pharmacy and Science, Tainan, Taiwan; 4 Department of Internal Medicine, Chi Mei Medical Center, Liouying, Tainan, Taiwan; 5 Department of Medicine, Chi Mei Medical Center, Tainan, Taiwan; Beijing Institute of Microbiology and Epidemiology, CHINA

## Abstract

In this study, we retrospectively reviewed the clinical experience of patients receiving doripenem-containing regimens for the treatment of healthcare-associated infections (HCAIs) in a tertiary care center and assessed the clinical usefulness of doripenem therapy in this clinical setting. In this retrospective study, the medical records of all adult patients who had ever received doripenem-containing therapy for the treatment of HCAIs were reviewed between September 1, 2012 and August 31, 2014, and the following data were extracted: age, gender, type of infection, disease severity, underlying comorbidities or conditions, and laboratory results. Additionally, we also extracted data regarding the rates of mortality and clinical and microbiological response. A total of 184 adult patients with HCAIs who had received doripenem-containing therapy were included in this study. Respiratory tract infections (n = 91, 49.5%) were the most common type of infection, followed by urinary tract infections, intra-abdominal infections and skin and soft tissue infections. The mean APACHE II score was 14.5. The rate of clinical success was 78.2%, and the overall in-hospital mortality rate was only 13.0%. Among patients, in-hospital mortality was independently and significantly associated with APACHE II score (odds ratio (OR), 1.2825; 95% CI, 1.1123–1.4788) and achieving clinical success (OR, 0.003; 95% CI, 0.0003–0.409). In conclusion, the overall in-hospital mortality rate was low and the clinical success rate was high among HCAI patients receiving doripenem treatment. These results suggest that doripenem may be judiciously used for the treatment of patients with HCAIs.

## Introduction

In an era of increasing bacterial antimicrobial resistance, broad-spectrum antibiotics, such as carbapenems, have been recommended as one of the most effective antibiotics in the treatment of serious infections caused by resistant pathogens, especially multidrug-resistant gram-negative bacilli (MDR-GNB) [1,2]. Within the carbapenem category, imipenem and meropenem are the most common used agents for healthcare-associated infections (HCAI), and ertapenem is indicated for the treatment of community-acquired infections. However, in response to increasing use of imipenem and meropenem, bacteria have increasingly demonstrated decreased carbapenem susceptibility [3,4]. In this context, doripenem—an agent in the carbapenem class that has been recently introduced into clinical use—may be another drug of choice due to exhibiting in vitro antimicrobial activity that is comparable with that of other carbapenems [5–9].

Because most automated susceptibility testing panels [10] do not include doripenem, and knowledge about the in vitro activity of doripenem against clinically isolated pathogens in the context of increasing carbapenem resistance is limited [11,12], clinicians usually more frequently prescribe imipenem or meropenem rather than doripenem in clinical practice. Thus, data regarding the clinical experience of patients receiving doripenem are limited. However, carbapenems other than imipenem and meropenem are needed for treating HCAIs, which are commonly caused by multidrug-resistant Gram negative bacilli (MDR-GNB). Currently, the only FDA-approved indication for doripenem is the treatment of adults with complicated intra-abdominal infections and complicated urinary tract infections. As a member of the carbapenem family, we would expect that further clinical usefulness of doripenem may be demonstrated. Therefore, we retrospectively reviewed the clinical experience of patients receiving doripenem-containing regimens for the treatment of HCAIs in a tertiary care center and assessed the clinical usefulness of doripenem therapy in this clinical setting.

## Materials and Methods

### Patients and hospital setting

This study was conducted in the Chi Mei Medical Center, a tertiary referral hospital that has 1288 beds. In this retrospective study, the medical records of all adult patients who had ever received doripenem-containing for the treatment of HCAIs were reviewed between September 1, 2012 and August 31, 2014. In this hospital, doripenem can only be prescribed after the approval of an infectious disease specialist according to the established guidelines. Additionally, patients who received doripenem for less than three days were excluded. The following data were extracted: age; gender; type of infection; disease severity, as indicated by Acute Physiology and Chronic Health Evaluation II (APACHE II) scores; underlying comorbidities or conditions, including cancer, stroke, liver cirrhosis, diabetes mellitus, congestive heart failure, chronic obstructive pulmonary disease, chronic respiratory failure on mechanical ventilation, chronic kidney disease, autoimmune diseases, use of steroids or immunosuppressants, HIV infection, and recent operations (within three months); and laboratory results. Additionally, we collected data regarding the rates of mortality and clinical and microbiological response. These data were collected on a routine basis, and analyses were carried out retrospectively. Therefore, no informed consent was required, and informed consent was specifically waived by the Institutional Review Board. Ethics approval was obtained from the Institutional Review Board of Chi Mei Medical Center.

### Definitions

Patients with HCAIs were identify based on having any one of the following risk factors: receipt of parenteral antibiotic treatment, chemotherapy or wound management within 30 days; living in nursing homes or long-term care facilities; being admitted to the hospital for more than two days within the prior 90 days; receipt of hemodialysis therapy in dialysis facilities; and clinical manifestations of infections requiring antibiotic treatment [13]. The diagnosis of the infection focus of the HCAI was made based on clinical, bacteriological, and radiological investigations as reported previously [14]. Mortality was defined as death from all causes during hospitalization. Similar to a previous report [15], a successful clinical response was defined as the resolution or improvement of the signs and symptoms of infection and requirement of no further antibiotic treatment after the discontinuation of doripenem therapy. In contrast, clinical failure was defined as the persistent presence of the signs and symptoms of the infection during doripenem treatment. Microbiological eradication was defined as the absence of the original baseline pathogens in a follow-up specimen.

### Statistical analysis

Continuous variables are reported as means and standard deviations, and categorical variables are presented as counts and proportions. The univariate differences between surviving and deceased patients at hospital discharge were examined using Student T tests or chi-square tests. A p value <0.05 was considered statistically significant. Statistical analysis was performed using SPSS 19.0 for Windows (SPSS, Inc., Chicago, IL, USA).

## Results

During the study period, a total of 184 adult patients with HCAIs who received doripenem-contain therapy were included in this study (Table 1) (data in S1 Appendix). Most of the patients received doripenem due to antimicrobial resistance (susceptibility-based; n = 165), while 15 received doripenem due to previously demonstrated drug intolerance, and one patient received doripenem because of an allergy to other antibiotics. A total of 27 patients received in combination with another antimicrobial agent. Among these patients, sulbactam was the most commonly used agent (received by 8 patients), followed by colimycin (received by 6 patients). The mean age of the patients was 72.4 years, and 145 (78.8%) patients were classified as elderly patients (≥ 65 years old). Men accounted for 61.4% of the patients. Respiratory tract infections (n = 91, 49.5%) were the most common type of infection, followed by urinary tract infections, intra-abdominal infections and skin and soft tissue infections. Approximately 45% of patients had various devices in place, such as a Foley catheter, central venous catheter, and endotracheal tube. Among the patients, the mean disease severity scores were 4.7 ± 3.1 according to their Charlson score, 4.2 ± 2.8 according to their SOFA score, 1.7 ± 1.5 according to their Pitt score, and 14.5 ± 5.9 according to their APACHE II score. Diabetes mellitus was the most common underlying disease, followed by cancer and stroke. Immunosuppressant and steroid use were identified in 7.6% and 29.3% of cases, respectively. *Pseudomonas aeruginosa* was the most common causative pathogen (n = 53, 28.8%), followed by *Escherichia coli* (n = 39, 21.2%), *Klebsiella pneumoniae* (n = 33, 17.9%) and *Acinetobacter baumannii* (n = 30, 16.3%). Table 2 shows the distribution of the eight most commonly detected organisms by sampling location. The distribution of varied in different types of clinical specimens. Extended spectrum beta-lactamase (ESBL) production was detected in 21 *E*. *coli* and 15 *K*. *pneumoniae* isolates. The average duration of doripenem use was 9.6 days. In terms of clinical response, 144 (78.2%) patients achieved clinical success after receiving doripenem-containing regimens. 50 patients who had the microbiologic response data, microbiologic eradication was only observed in 19 (38%) patients.

**Table 1:** Clinical characteristics of included patients.

Variables	Number (%) of patients (n = 184)
Age, years, mean ± SD	72.4 ± 13.4
Elderly patients	145 (78.8)
Male gender	113 (61.4)
Body weight, kg, mean ± SD	58.1 ± 13.0
Site of infection	
Respiratory tract infection	91 (49.5)
Urinary tract infection	48 (26.1)
Intra-abdominal infection	17 (9.2)
Skin and soft tissue infection	15 (8.2)
Primary bacteremia	6 (3.3)
Catheter-related infection	6 (3.3)
Meningitis	1 (0.5)
Device in situ	83 (45.1)
APACHE II score, mean ± SD	14.5 ± 5.9
Underlying diseases or conditions	
Diabetes mellitus	79 (42.9)
Cancer	67 (36.4)
Stroke	65 (35.3)
Gastric ulcer	49 (26.6)
Coronary artery disease	38 (20.7)
Chronic obstructive pulmonary disease	28 (15.2)
Congestive heart failure	16 (8.7)
End stage renal disease	15 (8.2)
Peripheral arterial occlusion disease	14 (7.6)
Liver cirrhosis	13 (7.1)
Hepatitis B	6 (3.3)
Hepatitis C	4 (2.2)
Autoimmune diseases	6 (3.3)
HIV infection	1 (0.5)
Steroid use	54 (29.3)
Immunosuppressant use	14 (7.6)
Alcoholism	1 (0.5)
Intravenous drug abuser	1 (0.5)
Recent operation (within three months)	59 (32.1)
Orthopedic implants	11 (6.0)
Pathogens	
*Pseudomonas aeruginosa*	53 (28.8)
*Escherichia coli*	39 (21.2)
*Klebsiella pneumoniae*	33 (17.9)
*Acinetobacter baumannii*	30 (16.3)
*Enterobacter cloacae*	11 (6.0)
*Proteus mirabilis*	6 (3.3)
*Citrobacter freundii*	5 (2.7)
*Pseudomonas stuartii*	5 (2.7)
Laboratory findings	
Procalcitonin, ng/mL, mean ± SD	12.8 ± 26.1
C-reactive protein, mg/L, mean ± SD	83.0 ± 81.4
BUN, mg/dL, mean ± SD	32.9 ± 32.0
Creatinine, mg/dL, mean ± SD	1.6 ± 1.6
Sodium, mmol/L, mean ± SD	136.7 ± 7.6
White blood cell, mean ± SD	13100 ± 11000
Hemoglobin, mean ± SD	10.4 ± 1.8
Platelet, mean ± SD	225200 ± 12600
Glucose, mean ± SD	161.1 ± 71.6
ALT, IU/L, mean ± SD	52.0 ± 77.0
AST, IU/L, mean ± SD	43.8 ± 33.4
Albumin, mg/dL, mean ± SD	2.5 ± 1.7
Duration of doripenem use, days, mean ± SD	9.6 ± 4.7
Clinical response	
Success	144 (78.2)
Failure	40 (21.7)
Microbiologic response*	
Eradication	19 (38.0)
Persistence	31 (62.0)
14-day mortality	22 (12.0)
30-day mortality	28 (15.2)
In-hospital mortality	24 (13.0)

*Microbiologic response data were obtained for 50 patients.

**Table 2:** Distribution of the eight most commonly detected organisms by sampling location.

Specimen	Number of isolates							
	*P*. *aeruginosa*	*E*. *coli*	*K*. *pneumoniae*	*A*. *baumannii*	*E*. *cloacae*	*P*. *mirabilis*	*C*. *freundii*	*Providencia stuartii*
Blood	8	10	4	4	5	2	1	0
Urine	10	19	4	2	3	3	3	4
Bile	2	1	2	0	1	0	2	0
Ascites	3	3	1	0	2	0	0	0
Respiratory specimen	36	3	21	24	3	2	0	1
Skin swab	6	4	3	4	1	0	0	0
Cerebral spinal fluid	1	0	0	0	0	0	0	0
Central venous catheter tip	1	0	0	0	1	0	0	0

Of the participants included in this study, 24 died, resulting in a mortality rate of 13.0%. Table 3 summarizes the results of the comparisons between the 24 deceased and 160 surviving patients. We found that deceased patients were more likely to have higher APACHE II scores, and underlying stroke than were surviving patients. In contrast, patients who achieved clinical success had a higher probability of survival to discharge than did patients with clinical failure (p < 0.001). Additionally, we found that the in-hospital mortality rate was highest among patients with respiratory tract infections (20.9%), followed by intra-abdominal infections (11.8%) and skin and soft tissue infections (6.7%). Among the four major types of HCAIs, the rate of clinical success was highest for intra-abdominal infections (94.1%), followed by urinary tract infections (89.6%), skin and soft tissue infections (80.0%) and respiratory tract infections (70.3%) (Table 4). Among the 50 cases for whom microbiological response data were available, the rates of microbiological eradication varied by type of infection. Moreover, Table 5 shows sites, clinical sources, and causal organisms of infections in the 31 cases who did not achieve microbiological eradication. Among the cases with microbiological failure, most of the clinical specimens were collected from drainage or devices, including biliary drainage, endotracheal tubes, Foley catheters, and external ventricular drainage. After performing multivariable analysis, we found that in-hospital mortality was independently and significantly associated with APACHE II score (odds ratio (OR), 1.2825; 95% CI, 1.1123–1.4788) and clinical success (OR, 0.003; 95% CI, 0.0003–0.409).

**Table 3:** Comparisons between deceased and surviving patients.

Variable	Number (%) of deceased patients (n = 24)	Number (%) of surviving patients (n = 160)	p value
Male gender	18 (75.0)	95 (59.4)	0.179
Elderly patients	21 (87.5)	124 (77.5)	0.302
Site of infection			0.050
Respiratory tract infection	19 (79.2)	72 (45.0)	
Urinary tract infection	2 (8.3)	46 (28.8)	
Intra-abdominal infection	2 (8.3)	15 (9.4)	
Skin and soft tissue infection	1 (4.2)	14 (8.2)	
Primary bacteremia	0 (0.0)	6 (3.8)	
Catheter-related infection	0 (0.0)	6 (3.8)	
Meningitis	0 (0.0)	1 (0.6)	
APACHE II score, mean ± SD	20.6 ± 7.1	13.6 ± 5.2	<0.001
Underlying diseases or conditions			
Diabetes mellitus	9 (37.5)	70 (43.8)	0.661
Cancer	8 (33.3)	59 (36.9)	0.8230
Stroke	14 (58.3)	51 (31.9)	0.014
Chronic obstructive pulmonary disease	6 (25.0)	22 (13.8)	0.217
End stage renal disease	4 (16.7)	11 (6.9)	0.113
Congestive heart failure	2 (8.3)	14 (8.8)	1.000
Liver cirrhosis	4 (16.7)	9 (5.6)	0.071
Autoimmune diseases	1 (4.2)	5 (3.1)	0.573
HIV infection	0 (0.0)	1 (0.6)	1.000
Steroid use	10 (41.7)	44 (27.5)	0.228
Immunosuppressant use	4 (16.7)	10 (6.3)	0.091
Intravenous drug abuser	0 (0.0)	1 (0.6)	1.000
Orthopedic implant	2 (8.3)	9 (5.6)	0.639
Duration of doripenem use, days, mean ± SD	9.6 ± 4.7	9.6 ± 4.5	0.607
Clinical response			<0.001
Success	1 (4.2)	143 (89.4)	
Failure	23 (95.8)	17 (10.6)	
Microbiologic response*			0.693
Eradication	2 (25.0)	17 (40.5)	
Persistence	6 (75.0)	25 (59.5)	

* Microbiologic response data were obtained for 50 patients.

**Table 4:** The rates of survival to discharge, clinical success and microbiologic eradication by primary site of infection.

Site of infection	Survival to discharge, n/N (%)	Clinical success, n/N (%)	Microbiological eradication, n/N (%)
Respiratory tract infection	72/91 (79.1)	64/91 (70.3)	7/24 (29.2)
Urinary tract infection	46/48 (95.8)	43/48 (89.6)	8/12 (66.7)
Intra-abdominal infection	15/17 (88.2)	16/17 (94.1)	1/5 (20.0)
Skin and soft tissue infection	14/15 (93.3)	12/15 (80.0)	1/5 (20.0)
Primary bacteremia	6/6 (100.0)	5/6 (83.3)	1/2 (50.0)
Catheter-related infection	6/6 (100.0)	5/6 (83.3)	1/1 (100.0)
Meningitis	1/1 (100.0)	1/1 (100.0)	0/1 (0.0)

**Table 5:** Sites, clinical sources, and causal organisms of infection among cases who did not achieve microbiological eradication.

Site of infection (case number)	Clinical specimens	Organism (number of isolates)
Respiratory tract infection (n = 17)	Sputum	*P*. *aeruginosa* (2), *A*. *baumannii* (2), *K*. *pneumoniae* (1)
	Endotracheal aspirate	*P*. *aeruginosa* (6), *A*. *baumannii* (4), *K*. *pneumoniae* (2)
Urinary tract infection (n = 4)	Catheterized specimen	*P*. *aeruginosa* (1), *Providencia stuartii* (1), *E*. *coli* (1), *K*. *pneumoniae* (1)
Intra-abdominal infection (n = 4)	Bile collected from biliary drainage	*E*. *coli* (1), *E*. *cloacae* (1), *P*. *aeruginosa* (1), *K*. *pneumoniae* (1)
Skin and soft tissue infection (n = 4)	Wound swab	*P*. *aeruginosa* (2), *A*. *baumannii* (2)
Primary bacteremia (n = 1)	Blood	*K*. *pneumoniae* (1)
Meningitis (n = 1)	External ventricular drainage	*P*. *aeruginosa* (1)

## Discussion

In this study, which included 184 patients with HCAIs, while we demonstrated the effectiveness of doripenem in the investigated clinical setting, the rates of clinical response and in-hospital mortality among included patients varied by type of infection. In the present work, respiratory tract infections were the most common indication for doripenem, with 91 respiratory tract infection cases receiving doripenem therapy, followed by urinary tract infections (n = 48), intra-abdominal infections (n = 17), and skin/soft tissue infections (n = 15). The overall rate of in-hospital mortality was 13.0%; however, the mortality rate ranged from 0% to 20.9% depending on the site of infection. Additionally, the clinical success rate was 89.4% and ranged from 70.3% to 100.0%. Our findings are in line with the findings of a review [16] of several clinical trials conducted by Lo et al. and a recent meta-analysis [17], both of which reported that doripenem was not inferior to the comparators, such as meropenem, imipenem, piperacillin/tazobactam, or levofloxacin, in its efficacy and safety profile in patients with a wide range of serious hospital-acquired infections. Thus, these results suggest that doripenem may serve as an effective treatment option in the armamentarium of antibiotics available to treat HCAIs.

For one of the FDA-approved indications, urinary tract infections, the rates of in-hospital mortality and clinical success were 4.2% and 89.6%, respectively, among the 48 nosocomial urinary tract infection patients. In addition, the rate of microbiological eradication was 75.5% (8/12). All four cases who did not achieve microbiological eradication had Foley catheters in situ, which may have been associated with increased difficulty in the eradication of causative pathogens. A Japanese study [18] demonstrated that the clinical efficacy of doripenem was comparable to that of meropenem in patients with complicated urinary tract infections (93.4% versus 92.4%) and that the two agents had similar bacteriologic response rates (95.9% in doripenem group versus 92.4% in meropenem group). The microbiological response to doripenem observed in the present work was similar to that reported in the study conducted by Vazquez et al. in which favorable microbiological responses were achieved in 71.4% of the 35 microbiologically evaluable patients receiving imipenem treatment for complicated urinary tract infection in a hospitalized adult population [19]. Therefore, both our findings and those of previous reports [16,18] suggest that doripenem may be as useful as other carbapenems for the treatment of hospital-associated urinary tract infections.

For another FDA-approved indication, intra-abdominal infections, the rates of in-hospital mortality and clinical success were 11.8%, and 94.1%, respectively, among the 17 hospital-acquired intra-abdominal infection cases. In a previous study that investigated the efficacy of tigecycline and imipenem in 199 patients with mild to moderately severe (mean APACHE II score of 4.6) complicated intra-abdominal infections, Chen et al. [20] found that the rate of clinical cure was 90.9%-97.9% among imipenem-treated patients and 81.7%-86.5% among tigecycline-treated patients. Another investigation [21] reported similar findings, suggesting that doripenem was effective and not inferior to meropenem in the treatment of complicated intra-abdominal infections (clinical cure rate: 77.9% to 85.9% versus 78.9% to 85.3%). In sum, these findings indicate that doripenem was as effective as other carbapenems and tigecycline for the treatment of intra-abdominal infections.

For healthcare-associated pneumonia, the rates of in-hospital mortality and clinical success were 20.9%, and 70.3% for 91 cases, respectively. Only a limited number of studies [22–24] have assessed the clinical effectiveness of doripenem for the treatment of nosocomial pneumonia. One prospective, randomized, open-label, multicenter study [22] found the clinical cure rate of nosocomial pneumonia following doripenem treatment was 81.3%, which was not different from the clinical cure rates observed following piperacillin/tazobactam treatment (79.8%). Another large, phase III study [23] compared doripenem with imipenem for the treatment of ventilator-associated pneumonia and that showed the clinical cure rates among clinically evaluable patients were 68.3% in the doripenem group and 64.2% in the imipenem group. Therefore, the authors concluded that doripenem was clinically efficacious and therapeutically non-inferior to imipenem in the treatment of ventilator-associated pneumonia [23]. However, one recent study [25] reported higher rates of clinical failure and mortality in microbiologically confirmed late-onset ventilator-associated pneumonia (VAP) patients who received a 7-day course of doripenem relative to those who received a 10-day course of imipenem. The difference observed in the VAP outcomes analysis may be due to differences in the duration of treatment (fixed 7 day course of treatment in the study conducted by Kollef et al. versus a longer course of 7–14 days in the study conducted by Chastre et al.). In our study, the mean duration of doripenem use was 9.6 days, and more than third-fourths of patients received more than 7 days of doripenem treatment. Thus, we observed better outcomes in patients with nosocomial pneumonia in the present work. In another Japanese study [24], a randomized, double-blind trial was conducted to compares doripenem with meropenem in the treatment of patients with respiratory tract infections. The authors found that the clinical efficacies were 92.7% in the doripenem group and 90.7% in the meropenem group, further confirming that doripenem is not inferior to meropenem in the treatment of respiratory tract infection [24]. In summary, although doripenem has not yet been approved by the FDA for the treatment of nosocomial pneumonia, the aforementioned findings and the results of our study both indicate that doripenem is not inferior to other carbapenems or beta-lactams in the clinical setting.

In this study, we found the clinical outcome of in-hospital mortality was independently associated with disease severity as indicated by APACHE II scores. This finding is reasonable and consistent with those of many previous studies [26–28], which indicated that higher disease severity contributes to overall mortality. This finding suggests that more aggressive treatment should be applied for the treatment of patients with more severe HCAIs. In addition, we found that the rate of clinical success was correlated with the rate of in-hospital mortality. Based on this finding, it may be inferred that the majority of in-hospital mortality occurred as a result of the HCAI itself; however, evaluating attributable mortality rather than all-cause mortality may help to further clarify this association.

There were several limitations to this study. First, this study was conducted in a referral center, and the number of included cases was limited; therefore, our findings may not be generalizable to other hospital settings. Second, this study was a retrospective investigation, and therefore, the results of this study may have been affected by different sources of bias, such missing data. However, for the most part, the impact of missing data on the study results was minimal. An exception the overall minimal bias associated with missing data may be observed in the evaluation of microbiological response. Only fifty patients had sufficiently complete data to allow for an assessment of the rate of microbiological eradication. Finally, we evaluated the clinical efficacy of doripenem in the treatment of HCAIs but did not assess the safety of doripenem-containing regimens. Further studies are warranted to clarify this issue.

## Conclusions

The most common indication for doripenem was respiratory tract infections, which is not an non-FDA approved indication, whereas the use of doripenem for FDA-approved indications, such as urinary tract infections or intra-abdominal infections, was less common in our institution. However, we found that the rate of overall in-hospital mortality rate was low and the rate of clinical success rate was high among HCAI patients. These results suggest that an adequate duration of doripenem treatment duration may be judiciously used for the treatment of patients with HCAIs, including respiratory tract infections and other FDA-approved indications.

## Supporting Information

S1 Appendix (XLSX)
